# Clinical Behavior of Bladder Urothelial Carcinoma in Young Patients: A Single Center Experience

**DOI:** 10.1155/2016/6792484

**Published:** 2016-08-02

**Authors:** Volkan Sen, Ozan Bozkurt, Omer Demir, Ahmet Adil Esen, Ugur Mungan, Guven Aslan, Aykut Kefi, Ilhan Celebi

**Affiliations:** Department of Urology, Dokuz Eylul University School of Medicine, 35330 Izmir, Turkey

## Abstract

*Background*. There is not enough evidence about clinical behavior of bladder cancer in younger patients.* Objective*. We aimed to evaluate the clinical characteristics and prognosis of bladder urothelial carcinoma patients under the age of 40 years.* Methods*. Medical records of patients listed in our cancer database were retrospectively reviewed. A total of 40 patients who were initially diagnosed with bladder urothelial carcinoma at the age less than 40 years were included in the study. Patients' records were reviewed for recurrence and progression rates, demographic data, medical history, and treatment modalities.* Results*. Pathological results revealed 33 (82.5%) Ta low-grade, 6 (15%) T1 high-grade, and 1 (2.5%) T2 high-grade urothelial carcinomas. Recurrence was detected in 14/39 (35.9%) patients but progression was not observed in any patients. The mean age of recurrent patients was significantly higher than nonrecurrent patients (34.8 versus 28.5 years; *p* < 0.05). Besides, recurrence was detected in only 1 patient with the age under 30 years (6.2%) and 13 patients (54.1%) between 30 and 40 years old, respectively (*p* < 0.05).* Conclusion*. Bladder urothelial carcinoma diagnosed at young age tends to be a low pathologic stage, with relatively low rate of recurrence and progression.

## 1. Introduction

Bladder cancer (BC) is the most common malignancy of the urinary tract [1]. More than 90% of bladder cancer is urothelial carcinoma and 70–80% of patients have non-muscle-invasive tumors at presentation [2]. Bladder urothelial carcinomas are more frequent in elderly patients with the median ages of initial diagnosis being 69 and 71 years in men and women, respectively [3, 4]. The clinical behavior of bladder urothelial carcinoma is a variable; the risk factors such as pathological stage and grade, presence of concurrent CIS, and tumor size and number constitute this variety. Although the risk factors and treatment modalities of bladder cancer have been well studied, conflicting reports exist about clinical behavior and prognosis for younger patients. This situation may be related to the rarity of bladder cancer in patients younger than 40 years old [5–8]. Some studies have reported that younger patients with bladder urothelial carcinomas had similar prognosis with older patients [9], but some of the studies showed that younger patients had better prognosis [10, 11]. We aimed to evaluate the clinical behavior and prognosis of bladder urothelial carcinoma patients under the age of 40 years.

## 2. Materials and Methods

Medical records of patients listed in our cancer database with diagnosis of bladder urothelial carcinoma between the years of 1990–2009 were retrospectively reviewed. A total of 40 patients who were initially diagnosed with bladder urothelial carcinoma at the age less than 40 years were included in the study. Data were recorded regarding their presenting symptoms, family history, and any exposure to occupational risk factors. Patients' records were also reviewed for recurrence and progression rates, demographic data, medical history, and treatment modalities. Tumors were staged according to the TNM staging system and graded according to the grading scheme proposed by World Health Organization (WHO) [12, 13].

Follow-up consisted of white light cystoscopy every 3 months for the first 2 years, every 6 months between second and fifth years, and then annually. Recurrence was considered as histological evidence of malignancies of the same or lower stage and grade. Tumor progression was defined as histologically confirmed muscle-invasive bladder cancer or evidence of metastatic disease.

### 2.1. Statistical Analysis

The statistical software package SPSS 20.0 version (SPSS Inc., Chicago, IL, USA) was employed for data management and analysis. Data were summarized with percentage, distribution, mean, standard deviation, and median values. Chi-square test was performed. Significance was set at *p* ≤ 0.05.

## 3. Results

A total of 40 patients were evaluated and median follow-up time was 54 (8–167) months. There were 7 (17.5%) women and 33 (82.5%) men. The mean age was 30.9 (10–39) years. The age distribution of patients was given in Table 1. The most common risk factor was smoking (25 patients; 62%). 33 (82.5%) patients had single tumor and 7 (17.5%) patients had tumors bigger than 3 cm. Pathological results revealed 33 (82.5%) Ta low-grade, 6 (15%) T1 high-grade, and 1 (2.5%) T2 high-grade urothelial carcinomas. Radical cystectomy was performed on only one patient. 16 patients were under the age of 30 and 24 patients were between 30 and 40 years old. Recurrence was detected in 14/39 (35.9%) patients but progression was not observed in any patients. Nine recurrent patients had primary Ta low-grade urothelial carcinoma and 5 recurrent patients had primary T1 high-grade urothelial carcinoma. The mean age of recurrent patients was significantly higher than nonrecurrent patients (34.8 versus 28.5 years; *p* < 0.05). Besides, recurrence was detected in only 1 patient with the age under 30 years (6.2%) and 13 patients (54.1%) between 30 and 40 years old, respectively (*p* < 0.05). The other possible predictive parameters for recurrence were similar between groups (Table 2).

## 4. Discussion

Bladder urothelial carcinoma is the most common malignancy of urinary tract; however, it is a very rare condition in young patients with the rate of 0.4% to 2.0% [5–8]. Because of its rarity there is a debate in the literature about the clinical behavior of bladder urothelial carcinoma in younger patients. Hematuria, the most common presenting symptom in bladder urothelial carcinomas in young patients [14], was detected in 85% patients (34/40) in our study. There was no significant difference between the recurrent and nonrecurrent patients regarding the occurrence of gross hematuria. Male/female ratio in our study was 4.7/1, similar to the literature [15]. Tobacco smoking is the most important risk factor for bladder urothelial carcinoma, accounting for approximately 50% of cases in the literature and 62% in our series [16, 17]. Bladder urothelial carcinomas in younger patients usually have a lower grade and stage than older patients [10, 18, 19]. With the increasing of age, the incidence of low-grade urothelial carcinoma decreases and high-grade urothelial tumor rate increases [20]. Our findings supported that the patients under 40 years old usually present with low stage and low-grade bladder cancer. Also younger patients have less recurrence and progression rates than older ones. Non-muscle-invasive bladder urothelial carcinoma was detected in 97.5% of our patients and only 1 patient had invasive tumor. Recurrence was detected in 14 (35%) patients but progression was not observed in any patients in our study. The comparison of patients aged <30 and 30–40 years revealed that recurrence was significantly higher in 30–40-year-old group. Our results and several published reports showed that tumor recurrence does seem to be age-related [21].

Although several tumor markers such as p53, pRb, HYAL-1, and nestin were evaluated for the prognosis of non-muscle-invasive bladder urothelial carcinomas, they could not take a place in routine clinical use [22–24]. Even if microsatellite instability and p53 were found to be associated with clinical behavior and prognosis of younger patients, further comprehensive studies including larger patient cohorts are needed to clarify this relationship [20, 25].

The prognosis of invasive bladder urothelial carcinoma in younger patients tends to be very poor. Yossepowitch and Dalbagni reported that the 5-year rate of disease-free survival after cystectomy was only 59% in 17 patients [19]. We performed cystectomy only in 1 patient for invasive bladder urothelial carcinoma and his survival was 24 months.

The main limitation of present study was its retrospective design. The staging, grading, and treatment modalities of bladder urothelial carcinoma had changed over years so the clinical outcomes might be affected by these changes in this period.

## 5. Conclusions

Bladder urothelial carcinoma diagnosed at young age tends to be a low pathologic stage, with relatively low rate of recurrence and progression. Therefore, more conservative management approaches in a long follow-up are needed for young patients with bladder cancer.

## Figures and Tables

**Table 1:** Age distribution of patients.

Age range (years)	*N* (%)
10–20	3 (7.5%)
20–30	13 (32.5%)
30–40	24 (60%)

**Table 2:** Clinical and pathological results of patients for different age groups.

	<30 years old	30–40 years old
Ta urothelial carcinoma	15 (93.8%)	18 (75%)
T1 urothelial carcinoma	1 (6.2%)	5 (20.8%)
T2 urothelial carcinoma	—	1 (4.2%)
Smoking	11 (68.7%)	14 (58.3%)
Recurrence rate	1/16 (6.2%)	13/24 (54.1%) **p** ** < 0.05**
Progression rate	—	—
